# District level estimates and mapping of prevalence of diarrhoea among under-five children in Bangladesh by combining survey and census data

**DOI:** 10.1371/journal.pone.0211062

**Published:** 2019-02-01

**Authors:** Sumonkanti Das, Hukum Chandra, Unnati Rani Saha

**Affiliations:** 1 Shahjalal University of Science & Technology, Sylhet, Bangladesh; 2 Indian Agricultural Statistics Research Institute (IASRI), New Delhi, India; 3 Department of Public Health, Erasmus MC, University Medical Center Rotterdam, Rotterdam, The Netherlands; Indraprastha Institute of Information Technology, INDIA

## Abstract

The demand for district level statistics has increased tremendously in Bangladesh due to existence of decentralised approach to governance and service provision. The Bangladesh Demographic Health Surveys (BDHS) provide a wide range of invaluable data at the national and divisional level but they cannot be used directly to produce reliable district-level estimates due to insufficient sample sizes. The small area estimation (SAE) technique overcomes the sample size challenges and can produce reliable estimates at the district level. This paper uses SAE approach to generate model-based district-level estimates of diarrhoea prevalence among under-5 children in Bangladesh by linking data from the 2014 BDHS and the 2011 Population Census. The diagnostics measures show that the model-based estimates are precise and representative when compared to the direct survey estimates. Spatial distribution of the precise estimates of diarrhoea prevalence reveals significant inequality at district-level (ranged 1.1–13.4%) with particular emphasis in the coastal and north-eastern districts. Findings of the study might be useful for designing effective policies, interventions and strengthening local-level governance.

## Introduction

Diarrhoea disease is the second leading cause of deaths in children under-five years old, and is responsible for killing around 525,000 children every year in the world [1]. Children who die from diarrhoea often suffer from underlying malnutrition, which makes them more vulnerable to diarrhoea. The incidence of diarrhoeal diseases is mostly common and a major public health problem in developing countries [2], where children under three years old experience on average three episodes of diarrhoea every year [3]. To improve the global child health, the UN has set a target under the sustainable development goal (SDG) 3 to end the epidemics of water-borne diseases and other communicable diseases by 2030 (target 3.3) with the aim of achieving the SDG target 3.2 of ending preventable deaths of children under-5 and of reducing under-5 mortality to below 25 per 1,000 live births [4]. Yet, another goal that targets to see a drop from diarrhoea to less than 1 in 1000 by 2025 (WHO 2013b).

According to the Bangladesh Demographic Health Survey (BDHS) 2014, it is observed that about 6 percent of children under-five years were reported to have had diarrhoea in the two weeks before the survey. This prevalence varied considerably across different geographical regions from as low as 2.7 in Rangpur division to as high as 6.5 in Barisal, 6.7 in Chittagong, 6.5 in Dhaka and 6.1 in Sylhet. In the recent past, Bangladesh has made a notable achievement in the development indicators of child health. Under-five mortality has declined in Bangladesh from 133 per thousand in the mid-90s to 46 in recent years [5,6]. The rate of stunting (low height compared to age) among under-five children, an indicator of the state of the chronic undernutrition in the population has come down from 55 percent in 1996–97 to 36 percent in 2014. On the other hand, the rate of wasting (low weight compared to height), an indicator of the state of the acute malnutrition in the population, is targeted to be below 5 percent by 2025 [1]. But, it still remains around 15 percent (15.6 percent in 2011 and 14.3 percent in 2014) [6]. Thus, if the UN target of child health is to be met it is essential to acceleration of reductions in the incidences of diarrhoea disease among children. Studies on determinants of diarrhoea diseases frequently report the risk factors such as child's age, sanitation facility, source of drinking water, hand wash, mother's education and place of urban-rural residence (see for example, Bado et al. [7]; Gebru et al. [8]). A prospective study shows that childhood diarrhoea prevalence is related with caregiver knowledge on the causes and prevention of diarrhoea [9]. Further, recent changes in the climatic factors including temperature, rainfall and salinity concentration increased the incidence of several infectious diseases including diarrhoea. Due to proneness to flood (middle and north-east part), drought (north-west part, particularly Rajshahi region) and salinity (coastal region at south) of different parts of Bangladesh, the episodes of diarrhoea are expected to vary over the country. A number of local studies in the flood (e.g., Manikganj, Shirajganj), drought (e.g., Rajshahi) and salinity (e.g., Satkhira, Potuakhali) prone areas have found a positive association between diarrhoea and climatic factors including temperature (heat wave and cold wave), rainfall (annual and seasonal) and salinity [10, 11].

Studies on child health often concern on increasing awareness about the problem and to quantify them at disaggregate level and to show the spatial inequity. Health planners and health practitioners require appropriate statistics at the level where programs are designed and implemented. BDHS report provides reliable estimates of diarrhoea prevalence at the national and divisional levels; however, due to lack in reliable estimates it masks the heterogeneity in the prevalence of diarrhoea at district level. To derive reliable estimates at the district level, the sample size in the demographic health survey is inadequate. Small sample size increases the sampling variability resulting in significant bias and errors of the estimates [12, 13]. The only source of district (or local or small area) level statistics are those that can be derived directly from census data, however, census data in Bangladesh do not cover child health indicators such as diarrhoea prevalence. Conducting a survey with aim to produce reliable district or small area level estimates is time-consuming and also costly. We, therefore, need special techniques that can generate reliable estimates at district or small area level utilizing the already available survey data. Small area estimation (SAE) is such a technique that can produce reliable estimates at small area level. The technique is a model-based method that links the variable of interest from survey with the auxiliary information available from census or administrative data sources for small areas. Depending on the availability of auxiliary information (covariates), small area models are of two broad types. One, the area level random effect model that is applied when the auxiliary information is available only at area level. This relates small area direct survey estimates to area-specific auxiliary information [14]. Two, the nested error unit level regression model, proposed originally by Battese et al. [15] that relates unit values of a variable to the unit-specific auxiliary information. We consider only the area level SAE method since covariates are available only at the area (or district) level. The standard Fay and Herriot method, based on the area level linear mixed model, is applicable to continuous outcome variables. However, the present analysis considers a different methodology, based on the area level version of logistic linear mixed model, where the target variable is binary with the auxiliary information available only at the area level. In this paper, we apply SAE technique to produce model-based estimates of diarrhoea prevalence among under-fives in different districts of Bangladesh. The SAE technique overcomes the sample size challenges and can generate representative and reliable estimates at the small area level by linking outcome of interests that are recorded in BDHS datasets with auxiliary data from census or administrative datasets. Here, small areas are defined as the districts of Bangladesh.

The SAE methods have been widely used in demographic, epidemiological, economic and social science researches [16–20]. The range of estimates at the small geographical level will provide us an insights at the level of inequality and inequity in the district level diarrhoeal prevalence. This information will enhance the capacity-building support where SDG target 17.18 emphasizes the need for disaggregated data for geographical location [4]. Most of the studies on analysis of diarrhoeal diseases in Bangladesh are based on hospital data or specific neighborhood level local data collected from some specific rural regions and hospitals conducted by ICDDR,B [21–22]. Also, the fitted models for determining the risk factors of child diarrhoea cannot be used for prediction due to unavailability of explanatory information in the census data. However, this study will generate the district level reliable and representative estimates of diarrhoea disease among under-fives in Bangladesh by exploiting the available information on diarrhoea episodes in BDHS 2014 and auxiliary information from census. The estimated diarrhoea prevalence will be mapped also to show spatial inequalities for visual representation and policy conclusion. The resulted conclusion of this study may help to reach at 2030 SDG target to strengthening capacity building support. The rest of the article is organized as follows. In section 2 we illustrate the data used for the analysis and in section 3 we present an overview of the SAE methodology used for the analysis. Section 4 introduces the diagnostic procedures for examining the model assumptions and validating the small area estimates, and describes the results from stakeholder point of view. Section 5 finally sets out the main conclusions.

## Data description and model specifications

In the SAE analysis, two types of variables are required. (i) the variable of interest drawn from the BDHS 2014 [6] for which small area estimates are required. The variable of interest for this study is the incidence of diarrhoea among children under-five years of age, which is binary at the unit level, corresponding to whether a child (under 5 years of age) had diarrhoea in the past 2 weeks preceding the survey or not. The parameter of interest is to estimate the proportion of children aged below 5 years with diarrhoeal disease (i.e. the incidence of diarrhoea) at small area (defined as the 64 districts of Bangladesh) level. (ii) The district level auxiliary (covariates) variables which are available in the Bangladesh Population and Housing Census 2011 [23]. The use of covariates from the Census 2011 to model incidence of diarrhoea among children under-five years of age from the 2014 BDHS raises the issue of comparability. However, the district level covariates used for our analysis are unlikely to vary much over a short period of time.

The Demographic and Health Surveys (DHS) program has been collecting demographic and health related data in Bangladesh since 1993/94 with a gap of approximately four years. The 2014 BDHS survey data is collected following a two-stage stratified sampling design (20 strata, 600 EAs, 30 households per EAs) covering all the 7 divisions and 64 districts. The completed 2014 BDHS data have 17,300 households, 17,863 ever-married women aged 15–49 years old, and 7,798 under-5 children [6]. Information on children diarrhoeal episodes is recorded from their mothers asking whether their children had experienced an episode of diarrhoea in the last two-week before the interview date. The number of eligible children for the study is 7560 of which about 6% children suffered from diarrhoea [6].

**Fig 1:**
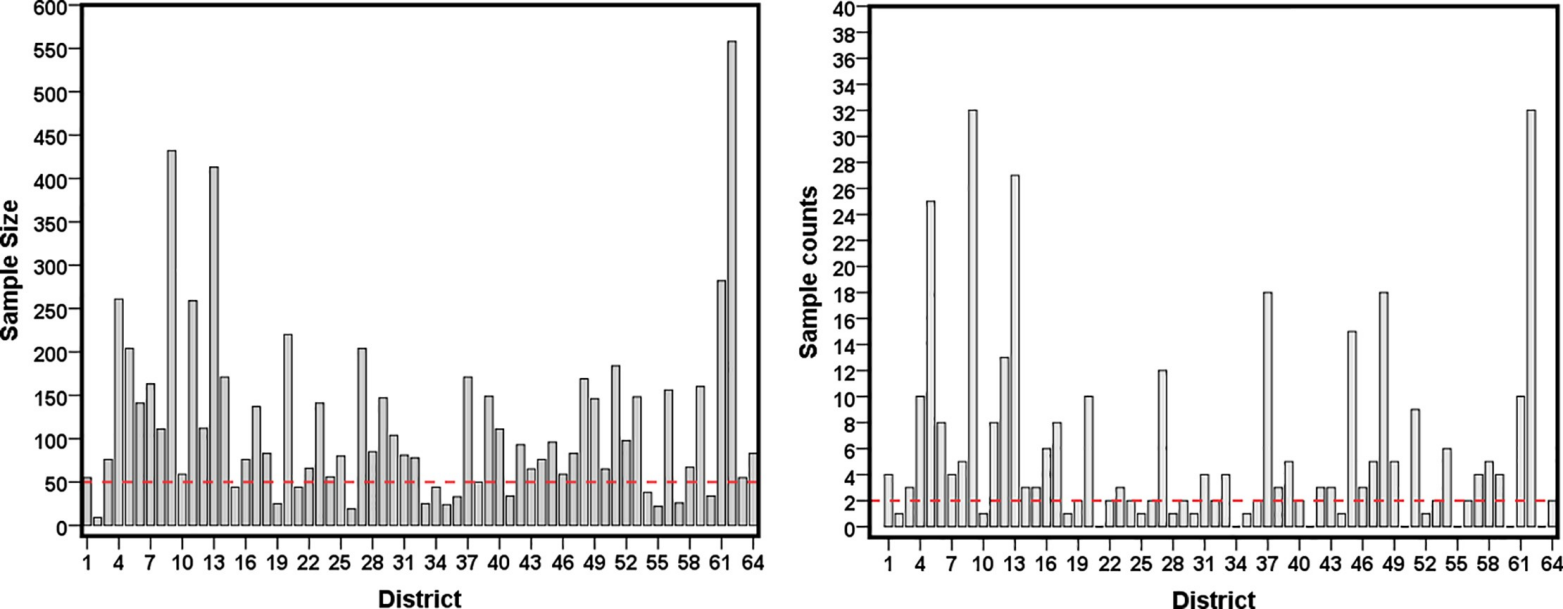
District-wise distribution of sample size (left hand side plot) and sample count (right hand side plot) in 2014 BDHS data.

Table 1 presents summary of sample size and sample count (i.e. number of diarrhoea incidence) in 2014 BDHS data which covers all 64 districts. Across districts, the sample size (i.e. number of under-five children) ranges from 9 to 556 with an average of 118. Fig 1 depicts the distribution of sample under-fives and diarrhoea incidence over 64 districts. It is clearly evident from Fig 1 that to derive direct district level estimates of diarrhoea incidence among under-fives are not possible due to small sample size. Out of 7560, the prevalence of diarrhoea (any types) during last two weeks was observed among 371 (only 5% unweighted) under-five children which is our primary interest of SAE analysis to derive district level estimates. The average sample count (occurrence of diarrhoea) per district was about 6 children with a minimum of 0 in some districts (7) and a maximum of 32 in two districts (see Table 1). It is observed that about 50% of total districts are sampled below 100 under-fives (left panel of Fig 1). The prevalence of diarrhoea is revealed to be very unequal over the districts (right panel of Fig 1). However, the distribution needs to be validated and statistically justified for policy conclusion. Therefore, our interest is to employ SAE technique to validate this descriptive distribution of diarrhoea prevalence. The resulted estimates would be important for policy planners and program managers to distribute resources in an effective way to improve health of under-fives in Bangladesh.

**Table 1:** Summary of sample size and sample count in 2014 BDHS data.

Characteristics	Minimum	Maximum	Average	Total
Sample size	9	558	118	7560
Sample count (diarrhoeal incidence)	0	32	6	371
Sampling fraction	0.00008	0.0012	0.0006	0.0491

The 2011 Census covers information on some important socio-demographic characteristics including age, sex, education, schooling, employment, disability and housing characteristics. The Bangladesh Bureau of Statistics published a number of official statistics at the disaggregated level. A number of such contextual variables at district level have been extracted from the reports of 2011 Census. As for example, population density, sex ratio, dependency ratio, illiterate female population and so on are available in the published report (Zila reports published by BBS). A number of such district level covariates that can be utilized for small area modelling. Here, we fit a generalised linear model between district-specific sample (unweighted) proportions of diarrhoeal prevalence and set of auxiliary variables. This model is fitted using the glm() function in R and specifying the family as “binomial” and the district specific sample sizes as the weight. Our main aim here is to build a good explanatory and predictive model based on the available auxiliary data. Finally, five auxiliary variables, viz. ChildU5 (Proportion of children under age 5), HHSize4 (Total HH members < = 4), Literacy (Literacy rate), Own.HH (Owend Tenancy) and depratio (Dependency ratio) that significantly explain the model, are identified for use in SAE analysis (see Table 2). The results in Table 2 show that all the five auxiliary variables viz. ChildU5, HHSize4, Literacy, Own.HH and depratio are strongly significant as a predictor for the diarrhoeal prevalence. Further, except for depratio, the effects of diarrhoea prevalence are negative for other auxiliary variables.

**Table 2:** Model parameters for the generalised linear models for diarrhoeal prevalence.

Parameters	Estimate	Standard Error	z value	Pr(>|z|)
Intercept	11.99064	2.82390	4.246	2.17e-05 ***
HHSize4	-0.05020	0.01192	-4.213	2.52e-05 ***
ChildU5	-1.02215	0.18738	-5.455	4.90e-08 ***
Literacy	-0.08479	0.01880	-4.509	6.50e-06 ***
Own.HH	-0.04129	0.00858	-4.812	1.49e-06 ***
Depratio	9.48107	2.15371	4.402	1.07e-05 ***
*AIC*	316.22			

*** *p* < 0.001

**Fig 2:**
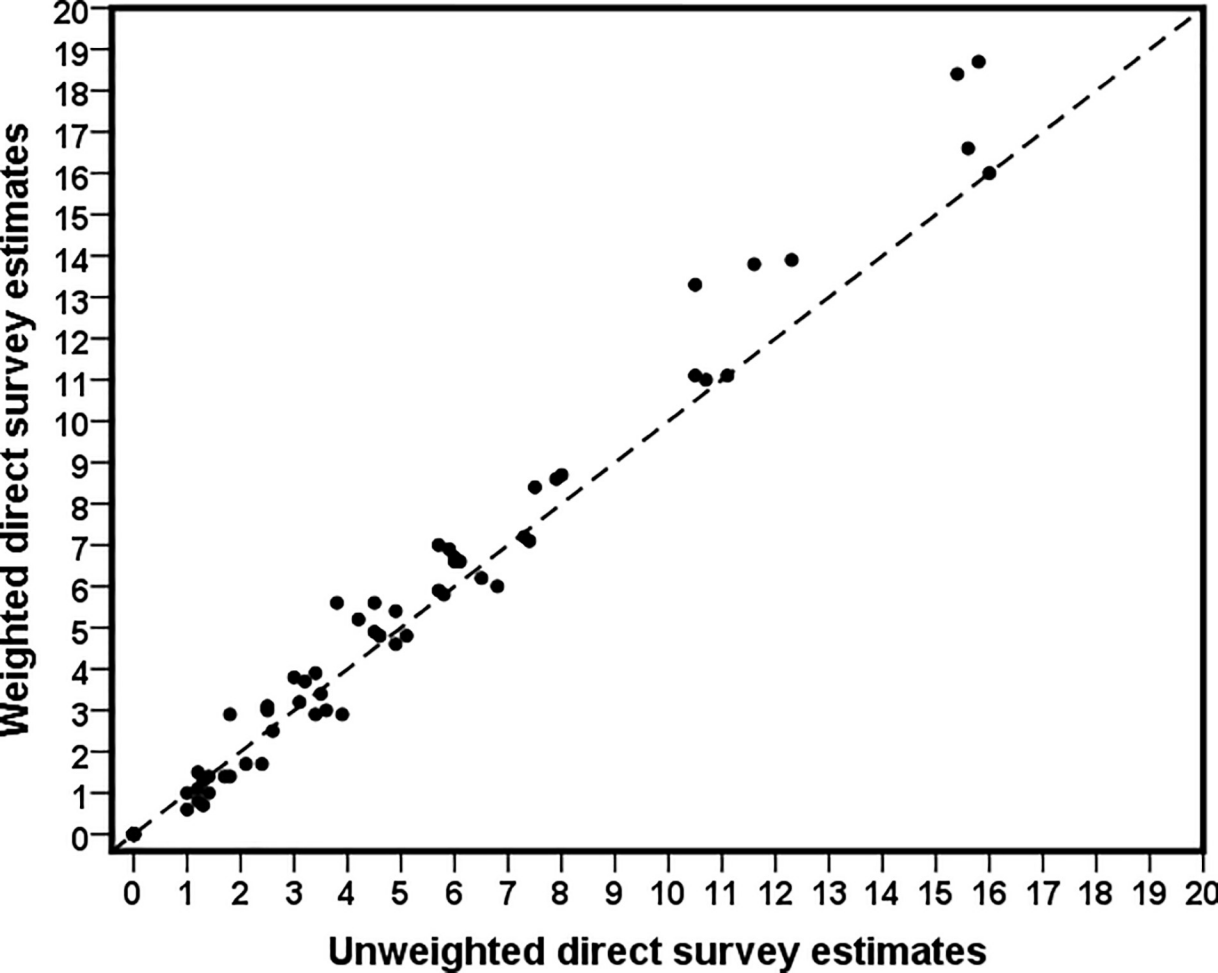
District-wise survey weighted vs unweighted direct estimates of diarrhoea prevalence (%) in Bangladesh.

**Fig 3:**
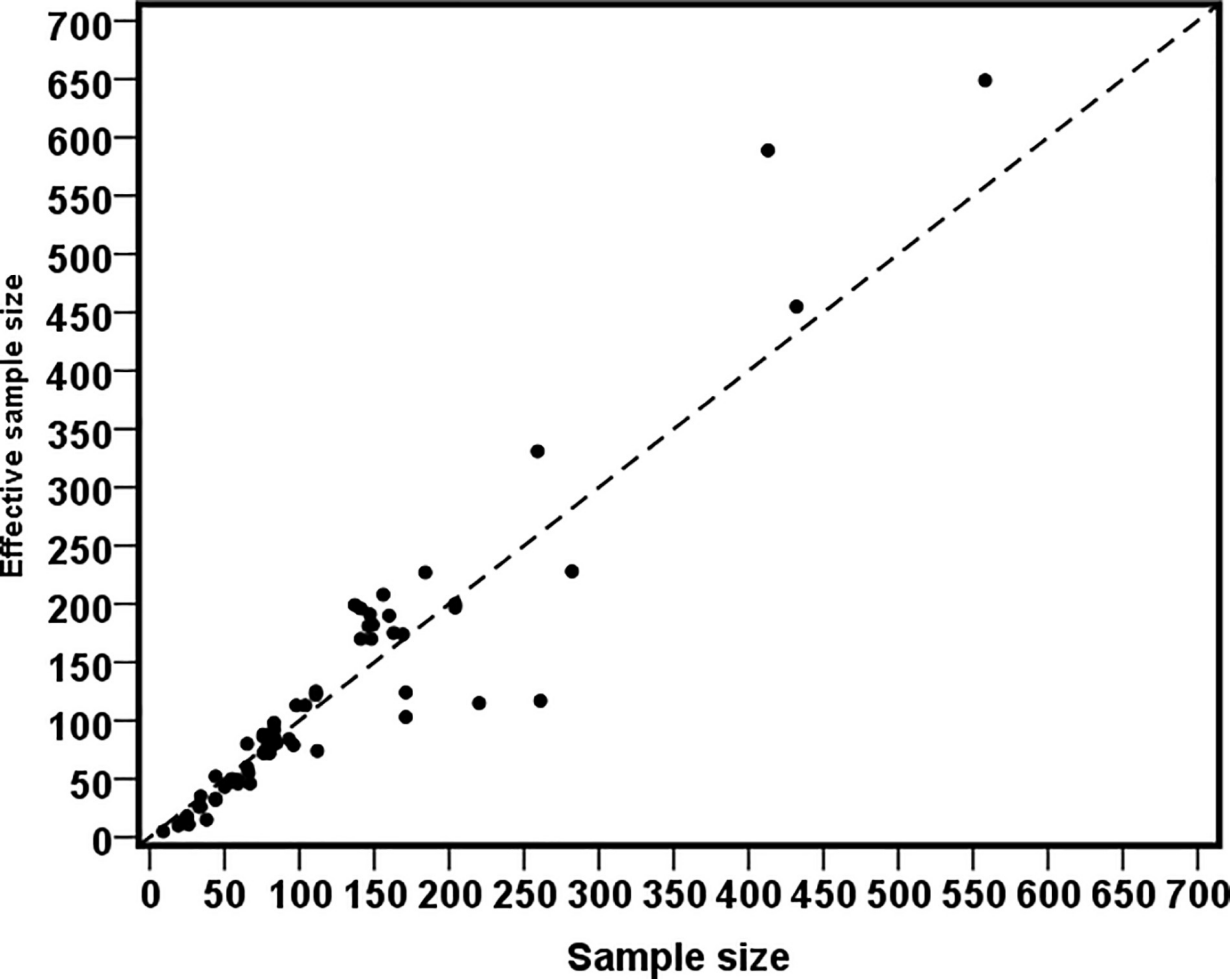
District-wise effective sample size vs observed sample size in BDHS 2014 data.

**Fig 4:**
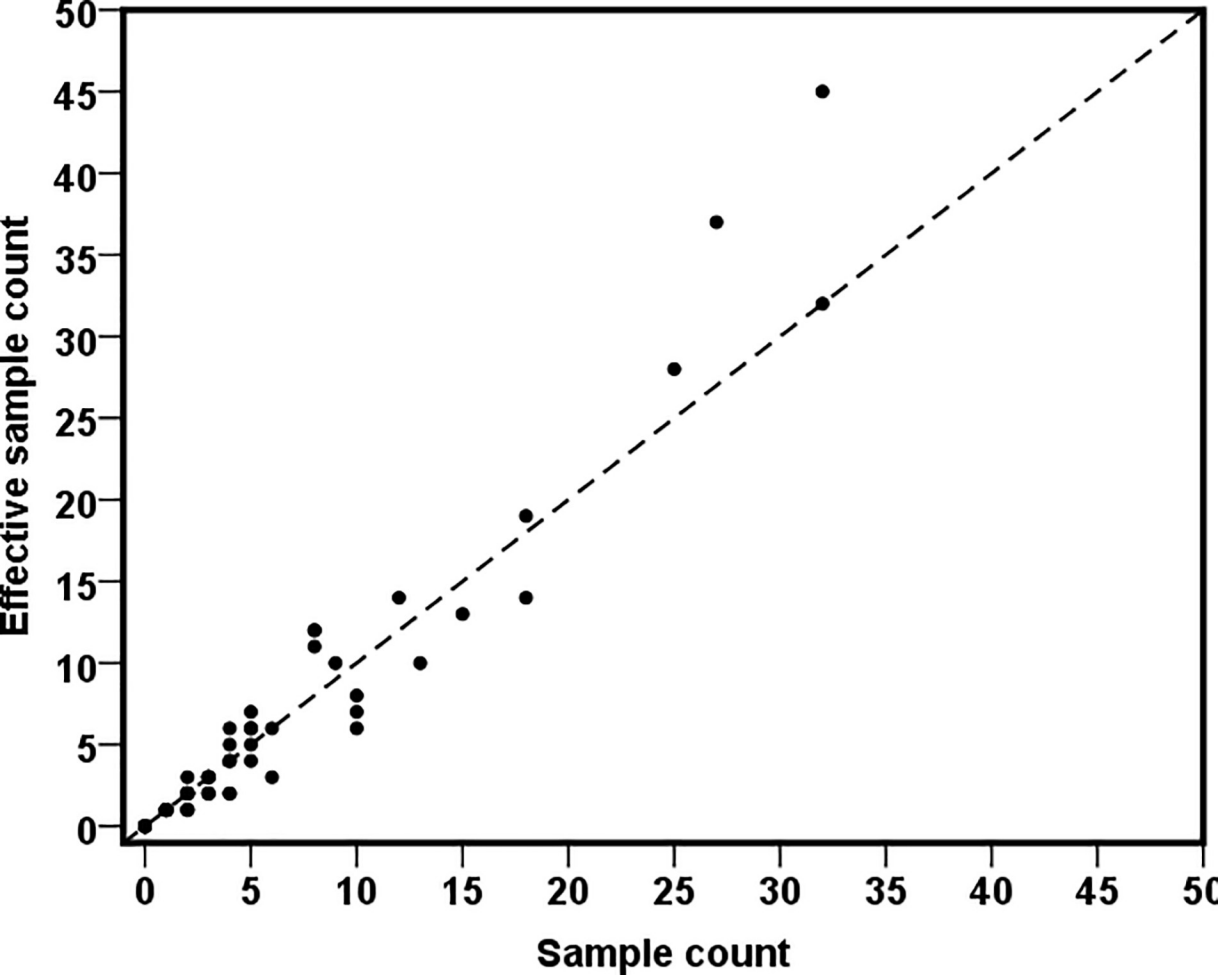
District-wise effective sample count vs observed sample count in BDHS 2014 data.

Fig 2 shows the district-wise survey weighted and unweighted direct estimates of diarrhoea prevalence (%) in Bangladesh, and reveals that sampling weights should not be ignored in the estimation otherwise it may underestimate the diarrhoea prevalence. This has the potential to seriously bias the estimates if the small area samples are seriously unbalanced with respect to population characteristics, and consequently use of the survey weights appears to be inevitable for if one wishes to generate representative small area estimates. Use of effective sample size rather than the actual sample size allows for the varying information in each area under complex sampling [24–25]. Fig 3 plots the effective sample sizes against the observed sample sizes. The effective sample counts (prevalence of diarrhoea) and observed sample counts are shown in Fig 4. In the majority of districts the effective sample size is larger than the observed sample sizes. Similarly, in most of the cases, the effective sample counts is larger than the observed sample counts. This indicates that the sampling design is informative, when compared with simple random sampling, in such districts. Hence, sampling weight cannot be ignored in SAE analysis.

## Methodology

Let us assume that a finite population *U* of size *N* consists of *D* non-overlapping and mutually exclusive small areas (or areas), and a sample *s* of size *n* is drawn from this population using a probability sampling method. We use a subscript *d* to index quantities belonging to small area *d* (*d* = 1,…,*D*). Following standard practice, we refer to these domains as small areas or just areas. Let *U*_*d*_ and *s*_*d*_ be the population and sample of sizes *N*_*d*_ and *n*_*d*_ in area *d*, respectively such that $U=\cup_{d=1}^{D}U_{d}$, $N=\sum_{d=1}^{D}N_{d}$, $s=\cup_{d=1}^{D}s_{d}$ and $n=\sum_{d=1}^{D}n_{d}$. We use subscript *s* and *r* respectively to denote quantities related to sample and non-sample parts of the population. Let *y*_*di*_ denotes the value of the variable of interest for unit *i*(*i* = 1,…,*N*_*d*_) in area *d*. The variable of interest, with values *y*_*di*_, is binary (e.g., *y*_*di*_ = 1 if child *i* under 5 years of age in area *d* has diarrhoea in the past 2 weeks preceding the survey and 0 otherwise), and the aim is to estimate the small area population count, $y_{d}=\sum_{i\in U_{d}}y_{di}$, or equivalently the small area proportion, $P_{d}=N_{d}^{-1}y_{d}$, in area *d* (*d* = 1,…,*D*). The standard direct survey estimator (hereafter denoted by DIR) for *P*_*d*_ is ${\hat{p}}_{d}^{Direct}=\sum_{i\in s_{d}}{\tilde{w}}_{di}y_{di}$, where ${\tilde{w}}_{di}=w_{di}/\sum_{i\in s_{d}}w_{di}$ is the normalized survey weight with $\sum_{i\in s_{d}}{\tilde{w}}_{di}=1$ and *w*_*di*_ is the survey weight for unit *i* in area *d*. The estimated variance of DIR is approximated by $v({\hat{p}}_{d}^{Direct})\approx \sum_{i\in s_{d}}{\tilde{w}}_{di}({\tilde{w}}_{di}-1){\left(y_{di}-{\hat{p}}_{d}^{Direct}\right)}^{2}$. See for example Särndal et al. [26] and Chandra et al. [27]. Under simple random sampling (SRS), the DIR is $p_{d}=n_{d}^{-1}y_{sd}$, with estimated variance $v(p_{d})\approx n_{d}^{-1}p_{d}(1-p_{d})$, where $y_{sd}=\sum_{i\in s_{d}}y_{di}$ denotes the sample count in area *d*. Similarly, $y_{rd}=\sum_{i\in s_{r}}y_{di}$ denotes the non-sample count in area *d*. If the sampling design is informative, this SRS-based version of DIR may be biased. Furthermore, DIR is based on area-specific sample data and can therefore be very imprecise when the area specific sample size is small or may even be impossible to compute if this sample size is zero. However, model-based SAE procedures that ‘borrow strength’ via a common statistical model for all the small areas can be used to address this problem [13]. If we ignore the sampling design, the sample count *y*_*sd*_ in area (district) *d* can be assumed to follow a Binomial distribution with parameters *n*_*d*_ and *π*_*d*_, i.e. *y*_*sd*_|*u*_*d*_~Bin(*n*_*d*_,*π*_*d*_). Similarly, for the non-sample count, *y*_*rd*_|*u*_*d*_~Bin(*N*_*d*_−*n*_*d*_,*π*_*d*_). Further, *y*_*sd*_ and *y*_*rd*_ are assumed to be independent binomial variables with *π*_*d*_ being a common success probability. This leads to *E*(*y*_*sd*_|*u*_*d*_) = *n*_*d*_*π*_*d*_ and *E*(*y*_*rd*_|*u*_*d*_) = (*N*_*d*_−*n*_*d*_)*π*_*d*_.

Let **x**_*d*_ be the *k*-vector of covariates for area *d* from available data sources. Following Johnson et al. [18] and Chandra et al. [27], the model linking the probability *π*_*d*_ with the covariates **x**_*d*_ is the logistic linear mixed model of form

$$logit(\pi_{d})=\ln\left\{\pi_{d}{\left(1-\pi_{d}\right)}^{-1}\right\}=\eta_{d}=x_{d}^{T}\beta +u_{d},$$ (1)

with $\pi_{d}=\exp(x_{d}^{T}\beta +u_{d}){\left\{1+\exp(x_{d}^{T}\beta +u_{d})\right\}}^{-1}$. Here **β** is the *k*-vector of regression coefficients, often known as fixed effect parameters, and *u*_*d*_ is the area-specific random effect that capture the area dissimilarities. We assume that *u*_*d*_ is independent and normally distributed with mean zero and variance $\sigma_{u}^{2}$. We can express the total population counts *y*_*d*_ as *y*_*d*_ = *y*_*sd*_+*y*_*rd*_, where the first term *y*_*sd*_, the sample count is known whereas the second term *y*_*rd*_, the non-sample count, is unknown. Under model (1), a plug-in empirical predictor (EP) of the population count *y*_*d*_ in area *d* is obtained as

$${\hat{y}}_{d}^{EP}=y_{sd}+\hat{E}\left(y_{rd}|u_{d}\right)=y_{sd}+\left(N_{d}-n_{d}\right)\left[\exp(x_{d}^{T}\hat{\beta }+{\hat{u}}_{d}){\left(1+\exp(x_{d}^{T}\hat{\beta }+{\hat{u}}_{d})\right)}^{-1}\right].$$ (2)

An estimate of the corresponding proportion in area *d* is ${\hat{p}}_{d}^{EP}=N_{d}^{-1}{\hat{y}}_{d}^{EP}$. It is obvious that in order to compute the small area estimates by Eq (2), we require estimates of the unknown parameters **β** and **u**. We use an iterative procedure that combines the Penalized Quasi-Likelihood (PQL) estimation of **β** and **u** = (*u*_1_,…,*u*_*D*_)^*T*^ with restricted maximum likelihood (REML) estimation of $\sigma_{u}^{2}$ to estimate unknown parameters [27–29]. Although PQL fitting in some cases may lead to inconsistent and biased estimators but this method works empirically well (Manteiga et al. [30]). The mean squared error (MSE) estimates are computed to assess the reliability of estimates and also to construct the confidence interval (CI) for the estimates. The expression for MSE estimate of empirical predictor (2) used in this analysis are given in Chandra et al. [27].

It is important to note that the model (1) is based on unweighted sample counts, and hence it assumes that sampling within areas is non-informative given the values of the contextual variables and the random area effects. The small area predictor based on (2) therefore ignores the complex survey design used in 2014 BDHS data. As noted earlier in Section 2, the sampling design used in 2014 BDHS is informative. Using the effective sample size rather the actual sample size allows for the survey weights under complex sampling. Furthermore, the precision of an estimate from a complex sample can be higher than for a simple random sample, because of the better use of population data through a representative sample drawn using a suitable sampling design. Following Chandra et al. [24], and Korn and Graubard [25], we model the survey weighted probability estimate for an area as a binomial proportion, with an “effective sample size” that equates the resulting binomial variance to the actual sampling variance of the survey weighted direct estimate for the area. Hence, in our analysis we replaced the “actual sample size” and the “actual sample count” with the “effective sample size” and the “effective sample count” respectively.

## Results and discussion

### Diagnostic measures

**Fig 5:**
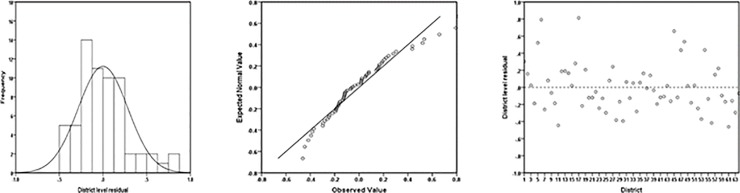
Histograms (left plot), normal q-q plots (centre plot) and distributions of the district-level residuals (right plot).

Generally, two types of diagnostics measures are suggested and commonly employed in SAE application; (i) the model diagnostics, and (ii) the diagnostics for the small area estimates [31]. The main purpose of model diagnostics is to verify the distributional assumptions of the underlying small area model, i.e. how well this working model performs when it is fitted to the survey data. The other diagnostics are used to validate reliability of the model-based small area estimates. In equation (1), the random area specific effects *u*_*d*_ are assumed to have a normal distribution with mean zero and fixed variance $\sigma_{u}^{2}$. If the model assumptions are satisfied, then the area (or district) level random effects (or residuals) are expected to be randomly distributed and not significantly different from the regression line *y = 0;* whereas, from Eq (1) the area (or district) level random effects (or residuals) are defined as ${\hat{u}}_{d}={\hat{\eta }}_{d}-x_{d}^{T}\hat{\beta }\left(d=1,\ldots ,D\right)$. The histogram and q-q plots are used to examine the normality assumption. Fig 5 exhibits the histogram of the district-level residuals, distribution of the district-level residuals and normal q-q plots of the district-level residuals. The plots in Fig 5 advise that the model diagnostics are fully satisfied with the data that we have used in this analysis. For example, Fig 5 shows that the randomly distributed district level residuals and the line of fit does not significantly differ from the line *y = 0*. The q-q plot as well as histogram also confirm the normality assumption.

For assessing validity and reliability of the model-based small area estimates (EP), we must use a set of diagnostics as described in Brown et al. [31]. These diagnostics are based on the argument that model-based small area estimates should be (a) consistent with unbiased direct survey estimates, i.e. they should provide an approximation to the direct survey estimates that is consistent with these values being "close" to the expected values of the direct estimates; and (b) more precise than direct survey estimates, as evidenced by lower mean squared error estimates, i.e. the model-based small area estimates generated by the EP $\left({\hat{p}}_{d}^{Direct}\right)$ should have mean squared errors significantly lower than the variances of corresponding direct survey estimates DIR [27, 32]. We consider four commonly used diagnostics measures that address these requirements, a bias diagnostic, a goodness of fit test, a percent coefficient of variation (CV) diagnostic, and a 95 percent confidence interval diagnostic. The first two diagnostics examine the validity and last two assess the reliability or improved precision of the model based small area estimates. In addition, we implemented a calibration diagnostic where the model-based estimates are aggregated to higher level and compared with direct survey estimates at this level [27, 32]. Here direct estimates DIR $\left({\hat{p}}_{d}^{Direct}\right)$ are defined as the survey weighted direct estimates.

We compute bias (Bias) and average relative difference (RE) between direct $\left({\hat{p}}_{d}^{Direct}\right)$ and model based $\left({\hat{p}}_{d}^{EP}\right)$ small area estimates as:

$Bias=D^{-1}\left(\sum_{d}{\hat{p}}_{d}^{Direct}\right)‑D^{-1}\left(\sum_{d}{\hat{p}}_{d}^{EP}\right)$, and $RE=D^{-1}\sum_{d}\left\{\frac{{\hat{p}}_{d}^{Direct}‑{\hat{p}}_{d}^{EP}}{{\hat{p}}_{d}^{Direct}}\right\}$ respectvely.

**Fig 6:**
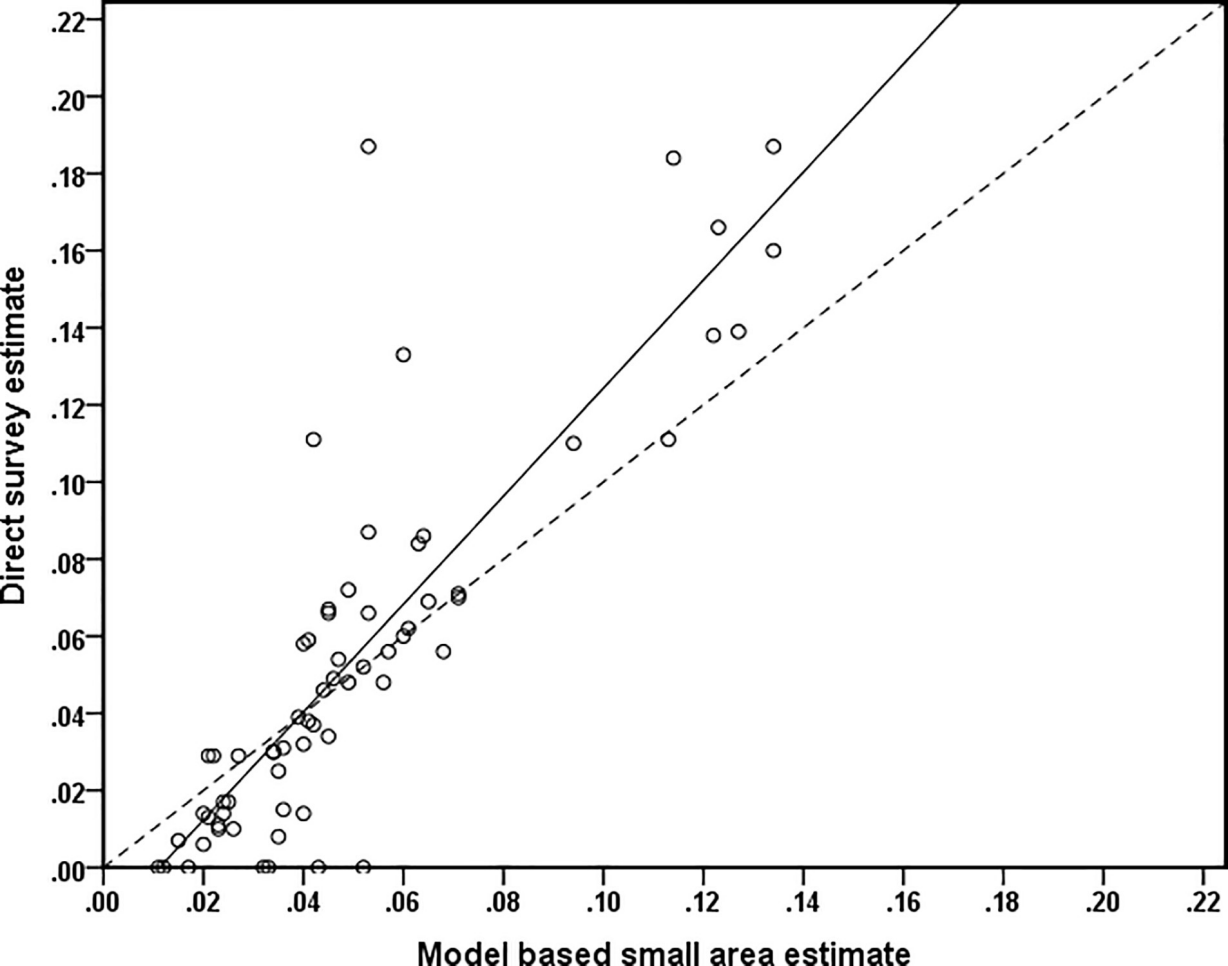
Bias diagnostic plot with y = x line (dotted) and regression line (solid) for diarrhoea prevalence (%) in Bangladesh.

The values of Bias and RE are 0.0044 and 0.579 respectively. We also apply Goodness of fit (GoF) diagnostic [31, 32], which is equivalent to a Wald test for whether the differences $Diff_{d}=({\hat{p}}_{d}^{Direct}‑{\hat{p}}_{d}^{EP})$ have a zero mean, and is computed as $W=\sum_{d}\left\{\frac{Diff_{d}^{2}}{var({\hat{p}}_{d}^{Direct})+mse({\hat{p}}_{d}^{EP})}\right\}$. The value of *W* is compared with an appropriate critical value from a chi-square distribution with degrees of freedom *D* equal to the number of Districts. In this analysis, *D* = 64, with a critical value of 83.675 at a 5% level of significance. We calculate *W* = 58.760 for this data, and so conclude that the model-based estimates are consistent with the direct estimates. Finally, in Fig 6 we provide bias diagnostic plot, defined by plotting direct survey estimates (*Y* axis) against corresponding model-based estimates (*X*-axis) and testing for divergence of the regression line from the *Y* = *X* line. This plot shows that the model-based estimates are less extreme when compared to the direct survey estimates, demonstrating the typical SAE outcome of shrinking more extreme values towards the average. The value of R^2^ for the fitted (OLS) regression line between the direct survey estimates and the model-based estimates is 73 per cent. Overall, these different bias diagnostics all show that the estimates generated by the model-based SAE method appears to be consistent with the direct survey estimates.

**Fig 7:**
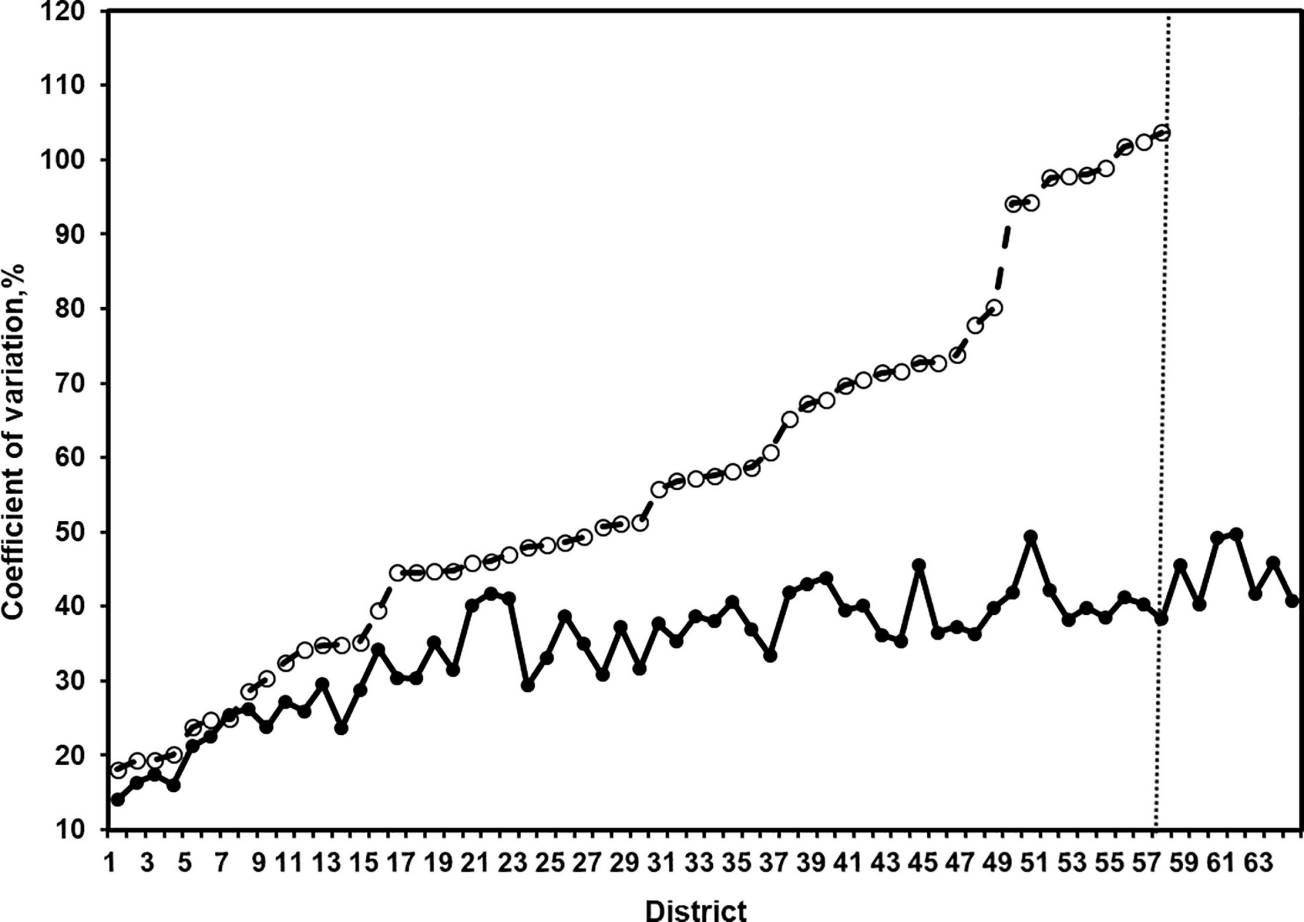
District-wise percentage coefficients of variation (CV, %) for the model-based small area estimate generated by EP (solid line, ●) and the direct estimate (dash line, ○) for the diarrhoea prevalence in Bangladesh. Districts are arranged in increasing order of CV of direct estimates.

A second set of diagnostics assess the reliability and improved precision of the model-based estimates relative to the direct survey estimates. The percent CV is the estimated sampling standard error as a percentage of the estimate. Small area estimates with large CVs are considered unreliable. There is no international standard for what constitutes "too large" in this context [18,19,27,32]. Table 3 provides district-wise values of the direct and the model-based estimates along percent CV and 95 confidence intervals. The distribution of percent CV of the direct and the model-based estimates plotted in Fig 7 shows significant improvement provided by SAE method. This indicates the improved precision of the model-based estimates when compared to the direct survey estimates. Fig 7 clearly reveals that in most of the districts, the CVs of the model-based estimates are significantly smaller than those of the direct survey estimates, implying that the model-based estimates vary less, and hence relatively more precise than the direct estimates. As one expects, the improvement in percent CV is higher for the districts with smaller sample sizes as compared to the larger sample sizes. For a few district (Bhola, Meherpur, Noakhali) with higher sample size (204, 171, 169) and higher diarrhoea prevalence (13.9, 11.1, 11.0), the difference in percent CVs is around 2%. While in some districts (Chuadanga, Gazipur, Joypurhat, Kushtia), there are more than 60% gain in percent CV with reasonable sample size (59, 83, 80, 104) but with only 1 diarrhoea prevalence (lower prevalence as a result shown in Table 3). Further, for the 7 districts (right hand panel in Fig 7), it is not possible to compute standard error and coefficient of variation for direct estimates because sample counts (diarrhoea incidence) for those districts are zero. However, this is the advantage of SAE technique that helps to predict the estimates even such districts with no sample information as well.

**Table 3:** Direct (DIR) and model-based (EP) estimates along with 95% confidence interval (95% CI) and percentage coefficient of variation (CV,%) of the diarrhoea incidence (%) by District in Bangladesh.

Division	District	DIR				EP			
		Estimate	95% CI		CV,%	Estimate	95% CI		CV,%
			Lower	Upper			Lower	Upper	
Barisal	Barguna	2.9	-0.4	6.2	58.21	2.2	0.4	4	40.66
Barisal	Barisal	5.6	0.3	10.9	47.99	6.8	2.9	10.7	29.41
Barisal	Bhola	13.9	8.6	19.2	19.42	12.7	8.4	17	17.43
Barisal	Jhenaidah	3	-1.3	7.3	72.75	3.4	0.4	6.4	45.56
Barisal	Patuakhali	4.6	1.5	7.7	34.26	4.4	2.2	6.6	25.91
Barisal	Pirojpur	0.6	-0.5	1.7	94.16	2	0.4	3.6	41.83
Chittagong	Bandarban	11.1	-9.4	31.6	94.36	4.2	0.1	8.3	49.37
Chittagong	Brahamanbaria	3.1	0	6.2	51.31	3.6	1.4	5.8	31.67
Chittagong	Chandpur	4.9	0.6	9.2	44.84	4.6	1.8	7.4	31.5
Chittagong	Chittagong	7.1	4.3	9.9	20.09	7.1	4.9	9.3	16.06
Chittagong	Comilla	3.2	1	5.4	34.88	4	2.1	5.9	23.72
Chittagong	Cox's Bazar	13.8	6.1	21.5	28.61	12.2	5.9	18.5	26.31
Chittagong	Feni	8.6	1.9	15.3	39.48	6.4	2.1	10.7	34.23
Chittagong	Khagrachhari	13.3	-3.7	30.3	65.24	6	1.1	10.9	41.83
Chittagong	Lakshmipur	5.4	0.2	10.6	49.35	4.7	1.5	7.9	34.96
Chittagong	Noakhali	11	5.9	16.1	23.82	9.4	5.5	13.3	21.28
Chittagong	Rangamati					5.2	1	9.4	41.69
Dhaka	Dhaka	6.2	3.9	8.5	19.28	6.1	4.1	8.1	16.39
Dhaka	Faridpur	6	-0.8	12.8	57.62	6	1.5	10.5	38.01
Dhaka	Gazipur	1.1	-1.1	3.3	101.77	2.3	0.4	4.2	41.25
Dhaka	Gopalganj	8.7	-2.8	20.2	67.29	5.3	0.8	9.8	43.03
Dhaka	Jessore	3.8	-1.4	9	70.46	4.1	0.9	7.3	40.08
Dhaka	Kishoreganj	1.5	-1.4	4.4	97.89	3.6	0.9	6.3	38.29
Dhaka	Madaripur	16	1.6	30.4	45.87	13.4	2.8	24	40.19
Dhaka	Manikganj	5.2	-4.8	15.2	97.63	5.2	0.9	9.5	42.13
Dhaka	Munshiganj	6.6	-0.6	13.8	55.72	5.3	1.4	9.2	37.74
Dhaka	Mymensingh	3.9	0.5	7.3	44.59	3.9	1.6	6.2	30.34
Dhaka	Narayanganj	3.7	-0.6	8	58.71	4.2	1.2	7.2	36.89
Dhaka	Narsingdi	4.8	-0.6	10.2	56.91	4.9	1.5	8.3	35.35
Dhaka	Netrakona	4.8	-0.6	10.2	57.27	5.6	1.4	9.8	38.71
Dhaka	Rajshahi	18.7	1.8	35.6	46.08	5.3	1	9.6	41.77
Dhaka	Satkhira	18.4	1.4	35.4	47.05	11.4	2.2	20.6	41.05
Dhaka	Sirajganj					3.3	0.3	6.3	45.96
Dhaka	Tangail					3.2	0.6	5.8	40.75
Khulna	Bagerhat	7.2	0.3	14.1	48.65	4.9	1.2	8.6	38.72
Khulna	Chuadanga	1.4	-1.4	4.2	102.48	4	0.8	7.2	40.31
Khulna	Jhalokati	1.7	-0.3	3.7	60.82	2.5	0.9	4.1	33.47
Khulna	Joypurhat	0.8	-0.8	2.4	103.67	3.5	0.9	6.1	38.33
Khulna	Khulna	6.9	2.8	11	30.31	6.5	3.5	9.5	23.83
Khulna	Kushtia	1	-0.9	2.9	98.96	2.6	0.6	4.6	38.46
Khulna	Magura					4.3	0.9	7.7	40.28
Khulna	Maulvibazar	6.6	-2.2	15.4	67.79	4.5	0.6	8.4	43.89
Khulna	Narail					1.7	0.1	3.3	49.22
Khulna	Shariatpur	8.4	0	16.8	51.12	6.3	1.7	10.9	37.23
Rajshahi	Bogra	5.9	1.8	10	35.22	4.1	1.8	6.4	28.86
Rajshahi	Jamalpur					1.2	0.1	2.3	45.64
Rajshahi	Naogaon	1.4	-0.6	3.4	73.84	2.4	0.6	4.2	37.27
Rajshahi	Natore	1.3	-1.2	3.8	98.04	2.1	0.5	3.7	39.84
Rajshahi	Nawabganj	16.6	8.5	24.7	24.93	12.3	6.2	18.4	25.45
Rajshahi	Pabna	2.9	0.2	5.6	48.26	2.7	0.9	4.5	33.13
Rajshahi	Rajbari	1	-0.5	2.5	77.84	2.3	0.7	3.9	36.38
Rajshahi	Sherpur	3	0	6	50.73	3.4	1.3	5.5	30.85
Rangpur	Dinajpur	2.9	-1.7	7.5	80.24	2.1	0.5	3.7	39.84
Rangpur	Gaibandha	5.8	1.8	9.8	34.82	4	1.7	6.3	29.58
Rangpur	Kurigram	1.4	-0.6	3.4	71.67	2	0.6	3.4	35.36
Rangpur	Lalmonirhat	2.5	-1	6	71.4	3.5	1	6	36.14
Rangpur	Nilphamari	6.7	0.8	12.6	44.73	4.5	1.4	7.6	35.14
Rangpur	Panchagarh					1.1	0	2.2	49.79
Rangpur	Rangpur	0.7	-0.3	1.7	72.79	1.5	0.4	2.6	36.51
Rangpur	Thakurgaon	1.7	-0.6	4	69.73	2.4	0.5	4.3	39.53
Sylhet	Habiganj	5.6	0.7	10.5	44.55	5.7	2.3	9.1	30.39
Sylhet	Meherpur	11.1	5.7	16.5	24.84	11.3	6.3	16.3	22.56
Sylhet	Sunamganj	3.4	1.2	5.6	32.51	4.5	2.1	6.9	27.22
Sylhet	Sylhet	7	4.5	9.5	18.16	7.1	5.1	9.1	14.08
**Summary Statistics of the Estimates**									
Minimum					18.16				14.08
Q1					39.48				30.37
Median					51.31				37.25
Mean					56.86				35.29
Q3					71.67				40.68
Maximum					103.67				49.79

**Fig 8:**
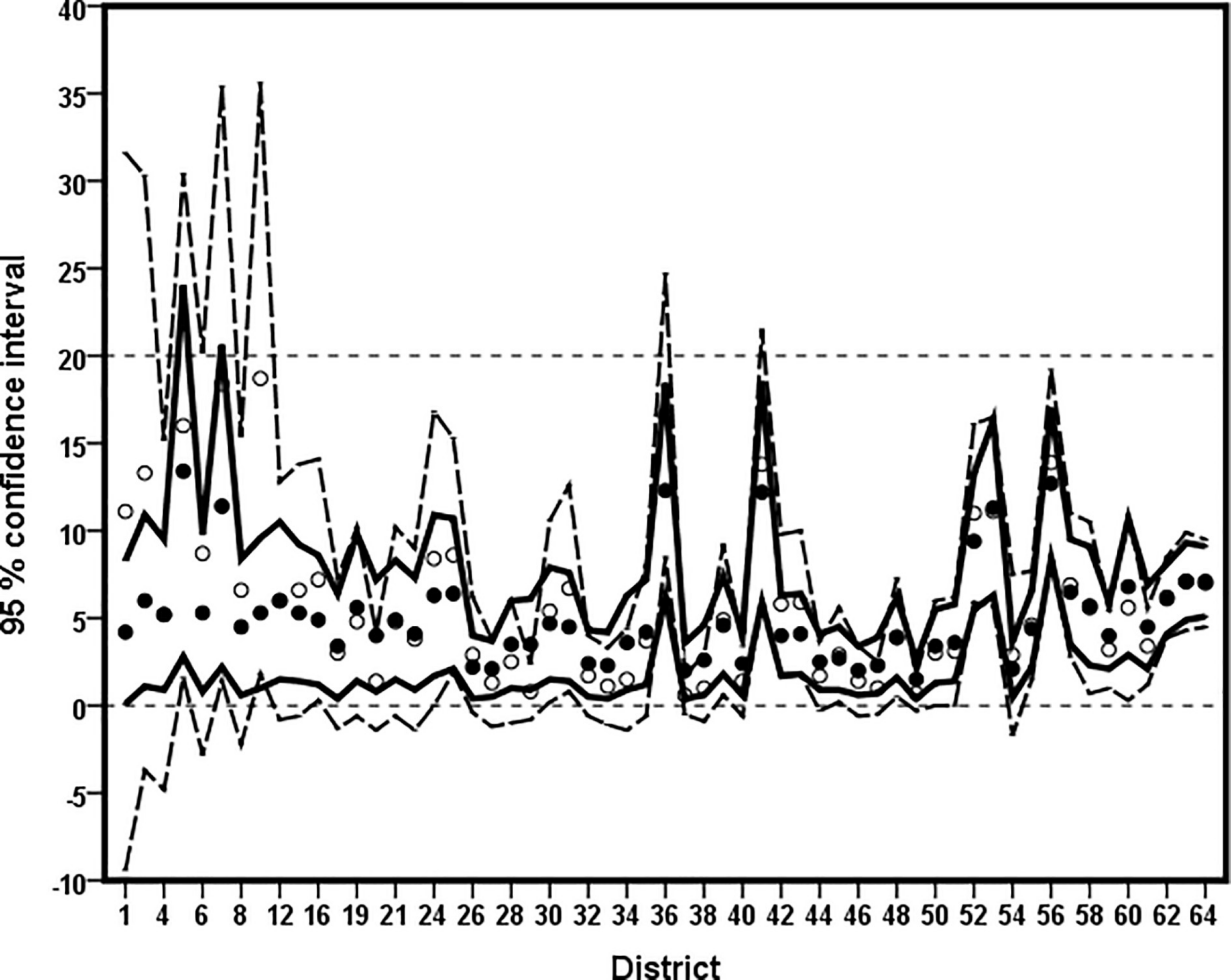
District-wise 95 percentage confidence interval (%95 CI) for the model-based small area estimate generated by EP (solid line, ●) and the direct estimate (dash line, ○) for the diarrhoea prevelance in Bangladesh. Districts are arranged in increasing order of sample size.

The 95% CI for direct estimates are invalid in many districts (see Fig 8) due to large standard errors. These are the districts with very small sample size or sample count. Further, for 7 districts with zero sample count, it is not possible to compute the standard error and hence % CV and 95% CI for direct estimates. In contrast, the model-based estimates of diarrhoea prevalence are still reasonable and representative for such districts. It is also clear that the direct and model-based 95% CI seems very close for the districts with reasonably larger sample size.

Finally, we investigate the aggregation properties of the model-based district-level estimates at higher (e.g. divisional level) level. Let ${\hat{P}}_{d}$ and *N*_*d*_ denote the estimate of proportion of diarrhoea incidence and population size for district *d*. The divisional-level estimate of the proportion of diarrhoea incidence is then calculated as $\hat{P}=\sum_{d=1}^{D}N_{d}{\hat{P}}_{d}/\sum_{d=1}^{D}N_{d}$. Bangladesh is divided into seven divisions, and aggregation properties can also be examined for these divisions. National and divisions level estimates of the proportion of diarrhoea incidence generated by the SAE method are reported in Table 4. Comparing these with the corresponding direct estimates we see that the model-based estimates are very close to the direct survey estimates at national level as well in each of the seven divisions.

**Table 4:** Aggregated level estimates of diarrhoea prevalence (%) generated by direct (DIR) and model-based (EP) method.

Estimator	National	Khulna	Dhaka	Sylhet	Barisal	Chittagong	Rajshahi	Rangpur
DIR	5.70	4.0	6.6	6.1	6.5	6.7	4.3	2.7
EP	5.23	4.1	5.3	6.6	6.7	6.4	4.0	2.5

### Spatial distribution of diarrhoea prevalence

**Fig 9:**
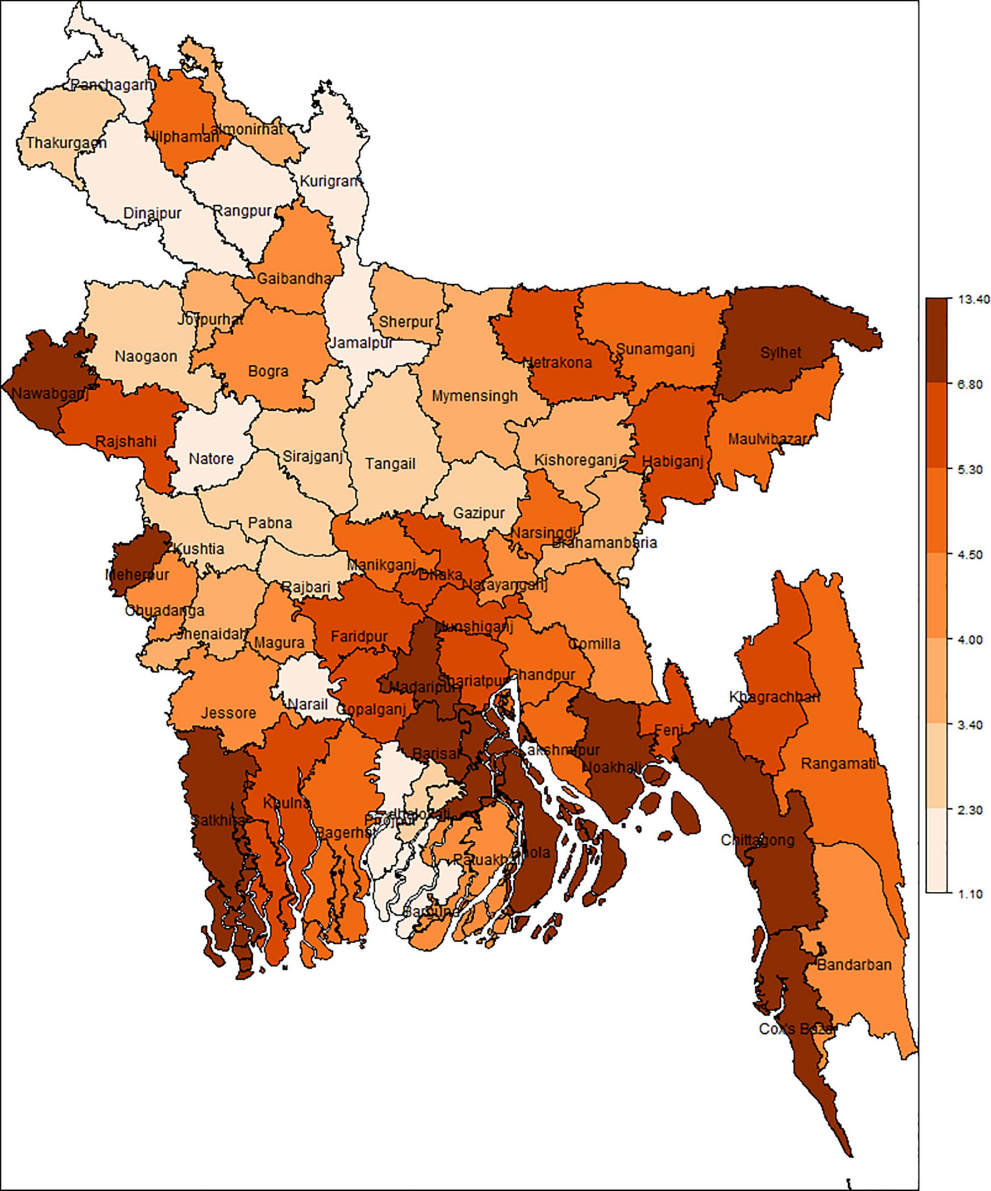
District-wise map showing the spatial distribution of diarrhoea prevalence (%) generated by model-based (EP) method in Bangladesh.

The results reported in Table 3 clearly show the degree of inequality with respect to distribution of diarrhoea prevalence in different districts in Bangladesh. The estimated prevalence of diarrhoea diseases among under-fives showing the spatial distribution are mapped in Fig 9. The map shows an unequal distribution of diarrhoea prevalence among under-fives in Bangladesh. The severity in diarrhoea incidence is observed more in coastal area and north-eastern part of Bangladesh ranged 4.60–13.40%. The prevalence of diarrhoea was observed more than the double of national level (6%) in Madaripur (13.4%), Stakhira (11.4%), Meherpur (11.3%), Bhola (12.7%), Cox’s Bazar (12.2%), and Nawabgonj (12.3%), see Table 3.

The result in Table 3 shows that there is a considerable variation in district level diarrhoea prevalence, even the prevalence of diarrhoea disease was observed more than the double of national level in some districts. The estimates in Table 3 and map in Fig 9 confirm a high degree of variation in diarrhoea prevalence at the district level. The prevalence of diarrhoea ranges from 1.1% in the Panchagarh district to 13.4% in the Madaripur district. The severity in diarrhoea incidence is observed more in the areas close to the water-porn areas, particularly in the southern coastal areas and north-eastern part of Bangladesh. The vulnerability in the north-eastern region is mainly due to frequent flash flood every year during the monsoon season. Also this area is known as haor where water stays a longer period of time after flood. The coastal region are also prone to salinity, which one of the main reasons of water-borne diseases like diarrhoea. The estimates (in Table 3) show that prevalence of diarrhoea is critical in the southern coastal and north-eastern districts of the country. For example, in Stakhira, Meherpur, Bhola, Cox’s Bazar and Nawabgonj districts, the estimates of prevalence of diarrhoea are 11.4%, 11.3%, 12.7%, 12.2% and 12.3% respectively. This clearly advises that a high proportion of children under five years of age across the southern coastal and north-eastern districts suffer from diarrhoea. In contrast, districts in the north-west part of the country, Jamalpur (1.2%), Rangpur (1.5%) and Narail (1.7%) are less prone to diarrhoea. This finding has policy implication and is in line with the distribution of the prevalence of malnutrition (measured as height-for-age) among under-fives in Bangladesh [33]. The district level estimates and mapping of prevalence of diarrhoea might be useful for policy guidance, resource allocation, and evaluation of development programme on hand washing, sanitation and safe drinking water. Besides, resulted conclusion may support SDG target 17.18 that emphasizes the need for disaggregated data for geographical location to strengthening capacity building support by 2020.

## Conclusions

Diarrhoeal disease is one of the leading causes of deaths in children aged under five years. Children living in poor or remote communities are most at risk and evidence shows children are dying from this preventable disease because of unequal and ineffective interventions across all communities. Designing effective intervention programs and monitoring strategies to reach “at risk” populations is a key concern for policy makers and program managers. WHO works with partner countries to promote national policies and investments that support case management of diarrhoeal diseases and their complications as well as increasing access to safe drinking–water and sanitation in developing countries (please see at http://www.who.int/en/news-room/fact-sheets/detail/diarrhoeal-disease).

Bangladesh committed to SDG of ending preventable deaths among under-fives aiming to reduce under-five deaths to 25 per 1,000 births by 2030. Therefore, exploring the vulnerable pockets is essential, which is what we study in this paper. Using SAE technique to link data from the Bangladesh DHS 2014 and Bangladesh population and Housing Census 2011, we have derived district level estimates of diarrhoea prevalence among under-5 children and mapped them to show the spatial inequality at district level. The results might be useful for the program managers and policy planners to implement their policy and interventions effectively. The use of the diagnostic measure e.g. coefficient of variation and the comparison with direct estimates confirm that the model-based district level estimates are robust and provide reliable district level estimates of diarrhoea prevalence. The study findings confirm that the national and regional level estimates of diarrhoea prevalence reported in the BDHS 2014 report mask the district level heterogeneity. Our study is the first that uncover the district level diarrhoea prevalence in Bangladesh with their accuracy measures.

## Supporting information

S1 File Original data set. District level direct estimates of diarrhea prevalence in Bangladesh obtained.
(XLSX)
